# An Ecological Conceptualization of Extreme Sports

**DOI:** 10.3389/fpsyg.2018.01274

**Published:** 2018-07-24

**Authors:** Tuomas Immonen, Eric Brymer, Keith Davids, Jarmo Liukkonen, Timo Jaakkola

**Affiliations:** ^1^Faculty of Sport and Health Sciences, University of Jyväskylä, Jyväskylä, Finland; ^2^Institute for Sport, Physical Activity and Leisure, Leeds Beckett University, Leeds, United Kingdom; ^3^Center for Sports Engineering Research, Sheffield Hallam University, Sheffield, United Kingdom

**Keywords:** extreme sports, ecological dynamics, phenomenology, affordances, form of life, perception and action

## Abstract

Currently, there are various definitions for extreme sports and researchers in the field have been unable to advance a consensus on what exactly constitutes an ‘extreme’ sport. Traditional theory-led explanations, such as edgeworks, sensation seeking and psychoanalysis, have led to inadequate conceptions. These frameworks have failed to capture the depth and nuances of experiences of individuals who refute the notions of risk-taking, adrenaline- and thrill-seeking or death-defiance. Instead, participants are reported to describe experiences as positive, deeply meaningful and life-enhancing. The constant evolution of emerging participation styles and philosophies, expressed within and across distinguishable extreme sport niches, or forms of life, and confusingly dissimilar definitions and explanations, indicate that, to better understand cognitions, perceptions and actions of extreme sport participants, a different level of analysis to traditional approaches needs to be emphasized. This paper develops the claim that a more effective definition, reflecting the phenomenology, and framework of an ecological dynamics rationale, can significantly advance the development of a more comprehensive and nuanced future direction for research and practice. Practical implications of such a rationale include study designs, representative experimental analyses and developments in coaching practices and pedagogical approaches in extreme sports. Our position statement suggests that extreme sports are more effectively defined as *emergent forms of action and adventure sports, consisting of an inimitable person-environment relationship with exquisite affordances for ultimate perception and movement experiences, leading to existential reflection and self-actualization as framed by the human form of life.*

## Introduction

Extreme versions of action and adventure sports, such as BASE jumping (including proximity flying), big wave surfing, rope-free solo climbing and big-mountain skiing or snowboarding, where death or severe injury is the most likely outcome of a mismanagement, mistake or accident, have been the subject of debate in various academic fields, e.g., psychology, sport science and outdoor studies.

In this perspective article, we propose a refined definition for ‘extreme sports’, specifically by looking at research on the most extreme versions of action and adventure sports, emphasizing a phenomenological approach. Preceding literature has explored extreme sports from a variety of frameworks and theoretical approaches, including sensation seeking (Rossi and Cereatti, 1993; Breivik, 1996), edgework (Laurendeau, 2006, 2008), type ‘T’ personality(Self et al., 2006) and psychoanalysis (Hunt, 1996). These ‘risk-centric’ approaches have offered only narrow and superficial views on extreme sports, mainly based on assumptions that participation reflects an innate need for thrills, excitement or adrenaline-seeking, and in many cases characterizing participation as pathological and unhealthy.

In recent years, a growing body of literature has argued that these are over-simplified explanations (e.g., Allman et al., 2009; Kerr and Mackenzie, 2012; Brymer and Schweitzer, 2013a; Monasterio et al., 2014). Conversely, it has been proposed that participation helps develop a profound person-environment relationship that can potentially offer a variety of ways for enhancing psychological and physical well-being and health (e.g., Kerr and Mackenzie, 2012; Brymer and Schweitzer, 2017; Frühauf et al., 2017). Phenomenological research has recently provided valuable insights, contrary to previous explanatory frameworks. For example, many participants are reported to describe positive lived experiences, refuting the thrill-seeking and adrenaline notion, instead characterizing participation as deeply meaningful and life-enhancing (Brymer and Schweitzer, 2013a, 2017; Holmbom et al., 2017). (see **Table 1**) In summary, due to varying approaches and conceptual definitions in the literature, discourse has been rather confusing. Furthermore, researchers have been unable to advance a consensus on what exactly constitutes an ‘extreme’ sport.

**Table 1:** Potential benefits of extreme sport participation.

(1) Increased positive psychological outcomes such as resilience, self-efficacy and positive affect	Brymer and Gray, 2009; Mackenzie et al., 2011; Brymer and Schweitzer, 2013a
(2) Opportunities to fulfill basic psychological needs of autonomy, competence and relatedness	Clough et al., 2016
(3) Opportunities to overcome challenge	Kerr and Mackenzie, 2012; Frühauf et al., 2017
(4) Opportunities to experience intense emotions	Brymer and Schweitzer, 2013a
(5) Increased physical activity levels	Gerber et al., 2012; Clough et al., 2016
(6) Experiencing integration with nature, i.e., *eutierria* (Albrecht, 2012, p.253)	Brymer, 2009; Brymer and Gray, 2009; Brymer and Schweitzer, 2015; Frühauf et al., 2017
(7) Contribution to deep friendships	Wiersma, 2014; Frühauf et al., 2017
(8) Positive transformational experiences	Allman et al., 2009; Brymer and Schweitzer, 2017; Holmbom et al., 2017

In our earlier work, we proposed ecological dynamics as a holistic, comprehensive framework for defining sports performance and for the study of participation in action and adventure sports (Immonen et al., 2017). In this position statement, we seek to recognize that, while extreme sports often share seemingly similar foundations or emerge from a similar history with other forms of constantly evolving action and adventure sport disciplines, they have become distinct activities with their own participation characteristics. For example, while BASE shares characteristics with other parachute sports it also has distinct equipment, skills, psychological characteristics and so forth from other non-extreme cousins. Thus, in similar vein to our earlier work, we include many aspects from the definition of action and adventure sports, adopting the broad concept of *affordances* (Gibson, 1979/1986) framed by the notion of a ‘form of life’ (Rietveld and Kiverstein, 2014). We emphasize that, while extreme sports may share similarities with some participation styles of action and adventure sports, they are not to be understood as similar disciplines or forms of life. We further narrow the scope of the discussion and conceptual definition of the most ‘extreme sports’, guided by phenomenological findings and fundamentally defined as: *emergent forms of action and adventure sports, consisting of an inimitable person-environment relationship with exquisite affordances for ultimate perception and movement experiences, leading to existential reflection and self-actualization as framed by the human form of life.*

In the following sections, we explain how understanding of participation in extreme sports can be enhanced through *the ecological dynamics* framework and conceptual understanding as led by phenomenology. We argue that an ecological dynamics rationale is useful because it can help us understand the emergence of complex, dynamical individual-environment systems in an extreme sports context. This framework provides an appropriate holistic framework, adaptable to zoom in on multiple perspectives and different time scales and levels of the person-environment system. This multidisciplinary and multidimensional viewpoint is necessary to make sense of the interacting constraints in extreme sports.

## Ecological Dynamics Enhances Understanding of Extreme Sports

Ecological dynamics has been influenced by ideas arising from complexity sciences, evolutionary biology, ecological psychology and non-linear dynamics (Davids et al., 2008, 2013a). It offers explanations for the rich patterns formed in complex adaptive systems with multiple components and levels, e.g., brain and behaviors of individuals or groups functioning at different timescales (Davids et al., 2008). A fundamental attribute in complex dynamical systems (such as a sport performer or community) is that they continuously adapt and change their organizational states in a non-linear fashion through harnessing system self-organization tendencies (Kelso, 1997; Davids et al., 2008). This is characterized by emerging coordination between system components or degrees of freedom and by synergetic relations between individuals and an environment (Bernstein, 1967; Renshaw et al., 2009; Chow et al., 2014). Importantly, coordination is understood as emerging in multiple dimensions, i.e., as motor coordination (coordinating limbs and body parts as degrees of freedom in relation to context) (Bernstein, 1967), but also at psychological, emotional and social levels.

Key ideas of ecological dynamics include the following: (a) behaviors are examined and understood at the individual-environment scale of analysis, (b) perception of information provides opportunities for action (i.e., affordances) and is the basis of how behaviors are regulated at an individual level, and (3), performance behaviors are self-organized over time under interacting constraints (Davids et al., 2008, 2013a; Hristovski et al., 2011; Seifert et al., 2017). In this view, the individual is seen as a component amongst an array of influential constraints within an individual-environment system (Chow et al., 2011). To understand behaviors or experiences of individuals, we need to expand the scope from emphasizing the innate needs or personality traits of individuals as explaining behavior or performances and, instead, understand their multi-dimensional relations with performance environments as inherent parts of the system.

### Constraints Shaping the Coordination of Behaviors

**FIGURE 1:**
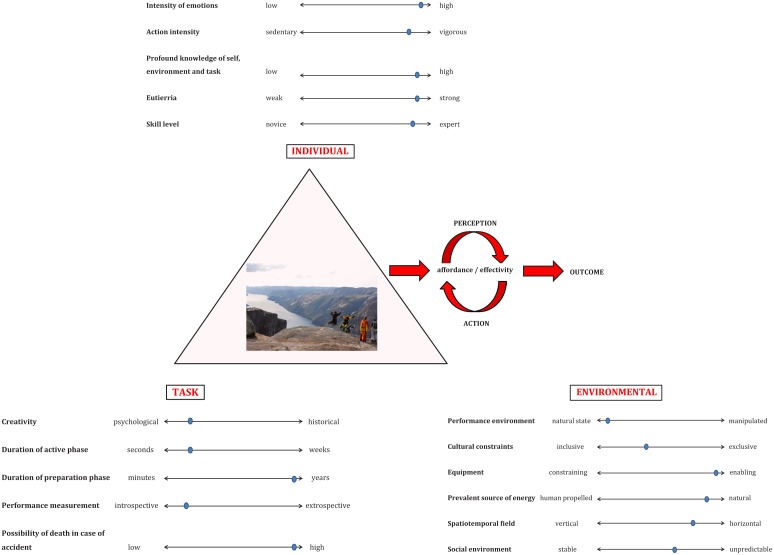
Derived from ideas of Newell (1986), Davids et al. (2008) and Immonen et al. (2017). Emerging, adaptive performer-environment relationships are scaled by situational and context-dependent key constraints that, in combination, define an extreme sport form of life/participation style. The comprehensive lens of Ecological dynamics is illustrated due to its capability to zoom into participation and engagement on multiple levels of analysis and time frames, reflecting extreme sport niches and performance outcomes as influenced and shaped by constraints from variety of levels. Due to the infinite combinations and complexity of interactions, irregular and unpredictable constraints are illustrated on continua, attending to significant situational and contextual fluctuations in each participant’s experience. To exemplify, exact positions on each continuum are depicted to illustrate the significance of each situational influencing constraint, which together as a whole consist the exemplary experience of a particular BASE jumper. For better understanding, new emerging constraints should be *a posteriori* included in the model according to complementary research findings and constant evolution of extreme sports. Included in the figure are constraints illustrating varying characteristics and performance environments of extreme sports in their current states of development and as reported in existing literature (e.g., Boden, 1996; Brymer and Oades, 2008; Brymer, 2009, 2010; Brymer and Gray, 2009; Thorpe, 2009; Breivik, 2011; Hristovski et al., 2011; Langseth, 2011; Varley, 2011; Albrecht, 2012; Kerr and Mackenzie, 2012, 2014; Davids et al., 2013b, 2016; Brymer and Schweitzer, 2013b, 2017; Wiersma, 2014; Yeh et al., 2015; Clough et al., 2016; Frühauf et al., 2017; Holmbom et al., 2017; Seifert et al., 2017). It is notable, that high embodied skill levels might not necessarily facilitate participation in extreme sports. Although attunement to affordances enabling behavior on the edges of one’s potential can be seen as a defining feature of the “extreme sport niche”, behaviors of individuals are subtly interconnected by multitude of interacting constraints. Image source: https://www.google.fi/search?as_st=y&tbm=isch&hl=fi&as_q=base+jumping&as_epq=&as_oq=&as_eq=&cr=&as_sitesearch=&safe=images&tbs=sur:f#imgrc=xfiYZa1hXCqt1M:

Constraints are boundaries or features shaping the emergence of each individual’s cognitions, actions and decision-making processes (Newell, 1986). Three categories of key constraints include *individual constraints*, which can be structural (e.g., height, weight, body shape, technical abilities, connectivity of synapses in the brain), historical (e.g., development to tolerate lack of comfort) and functional (e.g., motivations, emotions, cognitions, perceptions) characteristics of an individual (Davids et al., 2008, 2013a). In many traditional sports, such as football or cricket, influential *task constraints* are formed by specific rules, task goals and instructional features (Cordovil et al., 2009; Orth et al., 2014; Greenwood et al., 2016). This is a fundamental difference in comparison to most extreme sports, since they are most often free of organizational frameworks, regulated competitive structures and thus, rule-bound *task constraints*(Davids et al., 2013b; Immonen et al., 2017). *Environmental constraints* can be physical (e.g., weather, ambient light, temperature or gravity), or sociocultural (e.g., values, family or peer support, cultural norms) (Davids et al., 2008, 2013b). Adding to complexity, extreme sports take place in different kinds of environments, for example on land, in the air or on water (afloat or submerged) (Breivik, 2010; Davids et al., 2013; Immonen et al., 2017). For participants, it is crucial to become attuned to information in the environment by aligning coordination with natural conditions and sources of energy for action, such as the characteristics (tube, flat, etc.), size and speed of waves, currents and wind direction in big wave surfing. This is highlighted by the notion, that in any sport, but especially in extreme sports, constraints never remain truly fixed from one performance or session to the next (Rosalie and Müller, 2012). (**Figure 1**).

### Affordances Embedded in a Form of Life

James Gibson’s (1979/1986) concept of *affordances* has been widely discussed and debated in recent years, (e.g., Rietveld and Kiverstein, 2014; Rietveld et al., 2016; van Dijk and Rietveld, 2017; Withagen et al., 2017; Jordan et al., 2017). The widespread consensus contains the idea that affordances are relational ‘concepts’ combining features of the environment with individual *effectivities*, perceived on the basis of what they offer, invite or demand for an individual in terms of action possibilities. For instance, different surfaces, substances, objects or other individuals in the environment can afford different possibilities for different people in relation to individual constraints. *Effectivitie*s are complementary capabilities of an individual that can realize affordances in coherent forms of behavior (Shaw et al., 1982; Davids et al., 2016). Thus, the same mountain face or cliff can be seen as an affordance to play on a vertical plane for some climbers, to get airborne for BASE jumpers, or as a hazardous feature to avoid for recreational hikers. Also, in contrast to a novice, a skilled climber might perceive different kinds of opportunities (i.e., routes or moves) complementary to individual physical or psychological capacities due to learning and experience.

The Wittgensteinian concept of *‘form of life”* (Rietveld and Kiverstein, 2014) implies how sociocultural practices of humans can constrain the emergence of specific behavioral patterns. It is a key concept emphasizing that *effectivities* are not merely relative to particular individuals actually perceiving or detecting affordances, but also have an existence relative to the skills available in practice (an ecological niche), or, to the abilities available in a form of life as a whole. There cannot be a distinguishing discipline without the skills (relatively stable, collectively aligned perception-action couplings) defining it and enabling more affordances to be ‘revealed’ within, or further, beyond the sociocultural context (Immonen et al., 2017). Ingold (2000/2011) argues that much of what is called ‘cultural variation’ is in fact the variations of skills. To provide a practical example of skills relative to a specific (extreme vs. non-extreme) niche: a big mountain skier needs a mix of expert skiing skills and mountaineering skills. In addition, (at an extreme level) a deep knowledge of her/his personal constraints as well as constraints of the task and the environment are necessary, when adapting to a changing mountain terrain, weather and snow conditions or unpredictable social environments. In contrast, an urban freeskier might need a wide set of acrobatic manoeuvers and skills to shape the environment (e.g., building features from snow) to facilitate emergence of specific (aesthetic or original) styles of performance.

It might be useful and even necessary to acknowledge historical transformation patterns and influences, by including concepts such as *subcultures* or *lifestyles* (as types of constraints within a wider societal context) that can significantly shape participation of individuals. These notions alone, however, prove to be insufficient for the deeper examination of experiences, skill acquisition or evolution of niches. An essential notion here is that, although needed for ‘effective’ performance, high embodied skill level and expert knowledge (about self, task and performance environment) might not necessary lead to participation at extreme levels. Therefore, “An ecological niche is something that is available to a population of organisms, even if it is not completely used by any one member of that population” (Reed, 1996). It is useful to understand affordances and effectivities at the defining level of a form of life (i.e., the extreme sport niche), but acknowledge the level of individual engagement with regard to emerging experiences and performance outcomes.

Importantly, defining feature within extreme sport context is that one can also come to realize (under certain constraints) the potential to explore beyond a particular form of life, or sociocultural boundaries (Holmbom et al., 2017; Immonen et al., 2017). The relative lack of rule-bound constraints emphasizes an essential characteristic as indicated by historical development patterns of many extreme sports; the desire for ‘search’. This challenge is not limited only to search for functional solutions for motor problems (task) or exploration of new environments such as new mountain ranges, routes and climbing styles in mountain sports or greater depths in submerged sports. It also includes the exploration of personal capacities and profound (individual or shared) experiences or perceptions while the action unfolds (Davids et al., 2013b; Orth et al., 2017). Therefore, a distinguishing characteristic of extreme sports is that particular features can invite participants to function, while coordinating actions by challenging norms or ‘established’ movement patterns (Immonen et al., 2017).

### Evolution of Extreme Sport Niches Emerge Through Creative Exploration Under Interacting Constraints

Through closer examination, evolving extreme sport niches and individual performance outcomes are more complex than merely being subordinate to social norms or personality drives. They clearly involve individual tendencies to imagine, innovate and explore psychological capacities and possibilities outside of (affordances available to) a particular niche (Immonen et al., 2017). Psychological effectivities can thus support interactions of technical advancements and simultaneous exploitation of multiple affordances on many levels by an extreme sport athlete and lead to a new realization of the potential of human performance. Ingold’s (2000/2011) outlook on understanding embodied skills of practitioners as attributes of cultural variation introduces three fruitful levels of analysis to the notion of form of life. This is exemplified by the evolution of niches with a basis in the use of parachutes within a similar performance environment or spatiotemporal field: (1) Human forms of life in general, due to phylogenetic and ontogenetic development, do not possess the skill of flying (e.g., compared to birds or airborne insects); (2) There exist specific and distinguishing sociocultural practices, i.e., regularities in the skilled performances, behaviors and experiences of BASE-jumpers (extreme) as contrasted to sky divers (non-extreme) and (3) A more detailed, individual level of analysis indicates that skilled engagement with affordances is diverse, individualized and multi-dimensional within a specific sociocultural frame of reference. For instance, pioneers of proximity flying developed innovating wingsuits and airfoils to expand their body’s surface area. This allowed exploration and the opportunity to perceive the spatiotemporal field in a new way through different kind of forward motion, i.e., the ‘horizontal glide’ (Berry et al., 2010; Mei-Dan et al., 2013). This evolution (from ‘established’ styles of BASE-jumping toward proximity flying) illustrates the intertwined nature of constraints. That is, individuals’ experiences and performance outcomes emerge through interaction of underlying constraints on multiple levels. Simultaneously, the complex and non-linear evolution on all levels of the system (i.e., niches or disciplines) is inherently and bi-directionally dependent on the creative and exploratory behaviors of individuals.

Orth et al. (2017) proposed that, instead of a common proposition in cognitive science, where creative movement behaviors follow (or are an expression of) ideation (ideas represented in dedicated cognitive pathways), creative actions rather emerge while the action unfolds as a result of interactions amongst constraints. Importantly, creative actions are not, therefore, only products of individual constraints, but just as much emerge from interactions with task and environmental constraints. According to the ecological dynamics approach, this exploration is continuous and emerges over different timescales; not only when an action is produced, but also during practice and within and between practice sessions (Newell et al., 2001; Rosengren et al., 2003; Orth et al., 2017). Within complex adaptive systems, a state of *meta-stability* depicts when a system is poised between states of order and instability (Kelso, 2008). In the meta-stable state, fertile interactions can emerge spontaneously when previously uncorrelated system processes or components suddenly become interconnected under constraints (Chow et al., 2011). This is well illustrated by ‘the strapped crew’. The group of Hawaiian surfers explored action possibilities outside of the affordance landscape of traditional surfing. They experimented how to match the speed and size of big waves with the assistance of a jet-ski by combining their experiences and knowledge about different kinds of equipment as surfers, windsurfers and snowboarders. This emerging style of surfing became known as tow-in surfing, which continually set new big-wave records as new waves previously considered too big and fast to catch by paddling became possible to be surfed on smaller boards with bindings and a surfing style previously unseen (Warshaw, 2010). This advance in extreme sports exemplifies how a particular, ‘extreme’ style of surfing was not born solely in the minds of individuals. On the contrary, it emerged as a result of interaction of evolving constraints and participants’ exposure to landscapes of affordances on multiple domains. Through this expanded sociocultural frame of reference, broader landscape of affordances became available to be exploited during that particular era by individuals with adequate behavioral repertoire (Orth et al., 2018).

Through participating in various extreme sports or other non-extreme participation styles within action and adventure sports, which cross over, for example, in terms of equipment, environments or technical requirements, individuals are exposed to perceive multiple specific fields of affordances. Without this participation experience, more traditional exposure to sports may result in individuals inhabiting a narrow field in the affordance landscape. Additionally, technological advancements (e.g., affordance generation via social media) are constantly adding to the complex puzzle of psychological and social constraints that influence emerging individual-environment relationships. Thus, extreme sport niches in their current states of development should be seen only as ‘temporal emergent products’ of development in each area of individual, task and environmental constraints, yet open to constant evolution through creative exploratory behaviors. Therefore, it would be, at best, incomprehensive to define extreme sports without considering emerging creativity (meta-stability of the person-environment system) as an essential element.

## New Definition for Extreme Sports as Guided by Phenomenology

Human beings, as individuals within a form of life, engage the world in different ways. To understand the complex relationships of affordances and individual-environment systems, phenomenology is essential (Withagen et al., 2017). Phenomenology considers human consciousness as intentional, which means that consciousness and cognition is always directed at something (Brymer and Schweitzer, 2017). *Intentionality* implies that parts can only be understood against the background of wholes and objects against the background of their horizons. That is, the experiences or perceptions of extreme sport participants cannot be comprehensively explored and understood without the notion of the underlying and intertwined constraints i.e., the context. Phenomenology focuses on the explanations of experiences as lived, beyond cultural or psychological boundaries (Brymer and Schweitzer, 2017). The *lifeworld* is another core doctrine of phenomenology (Husserl, 1970; Crease, 2012; Husserl, 2012). Humans ‘seek’ authenticity, companionship, happiness or pleasure amongst numerous other things and they do this in a variety of ways, e.g., as children, parents, teachers or, athletes. This illustrates different ways of being, i.e., modifications of an array of the practical connections human beings have to the world. This array is defined as the *lifeworld.* Importantly, the way of experiencing the world depends not only what one’s environment in fact materially consists of, but also what is salient to an individual (Crease, 2012). As discussed earlier, this salience depends on relational properties (affordances) within a form of life. It again emphasizes the need to expand the scope of examination on extreme sports from strictly individual characteristics toward lifeworlds as individual compositions of the broader person-environment system.

Since many conceptual definitions influenced by traditional theory-led perspectives (e.g., Rossi and Cereatti, 1993; Self et al., 2006; Laurendeau, 2008) have led to interpreting extreme sport participation as pathological, socially unacceptable or unhealthy, the paradigm shift from risk-centric approaches toward more nuanced and comprehensive understanding of extreme sports has been mainly initiated by insights from phenomenological exploration of participants’ experiences (e.g., Willig, 2008; Breivik, 2011; Brymer and Schweitzer, 2017). Findings from phenomenological exploration of participants’ lifeworlds have shown that the extreme sport experience has the potential to dramatically change ways of being-in-the-world (Heidegger, 1996). For example, the profound person-environment relationship developed in extreme sports participation, can act as a facilitator to a deep, positive understanding of self and its place in relation to the environment (Brymer and Gray, 2009) i.e., *eutierria* (Albrecht, 2012). Experiencing intense fear can be a potentially meaningful and constructive event in the lives of participants, having implications for understanding fear as a potentially developmental and transformative process (Brymer and Schweitzer, 2013a). Participation can be a form of exploration of the ways in which humans experience fundamental human values (Brymer and Schweitzer, 2013b). Heidegger (1996) thought that a core aspect of being a human is our capability to make new worlds. Through the notion of form of life, extreme sports can be seen as a ‘world’ in similar fashion to the worlds of architecture or music, offering multiplicity of ways of being-in-the-world. This notion expands the perspective to broadly understand and define extreme sports, not solely as a pastime or ‘sports’ in a traditional sense, but as a type of form of life specific to being human. That is, as ways for humans to engage with the environing world and as mediums to explore physical or psychological capacities and, ultimately, what it means to be a human. Therefore, rather than a causal property of innate need, personality trait or any other single entity or component within the person-environment system, the extreme sport experience can be seen as a pure embodiment of the context, i.e., the composition of interacting, omnipresent and intertwined constraints (Jordan et al., 2017).

## Future Questions

Previous research has shown the evident link between affect, emotions and decision making (e.g., Blanchette and Richards, 2010; Angie et al., 2011). Studies have also indicated that in extreme sports, psychological factors such as fear, anxiety, excitement and pleasure, as well as beliefs and motivations, have significant roles during participation and a strong influence on how the environment is perceived by individuals (e.g., Sanchez et al., 2010; Lawrence et al., 2014). To better understand the relationship of decision making, affective constraints and ‘invitational character’ or ‘affective salience’ of affordances, more research is needed on the social dynamical systems within the extreme sport context. For example, research has indicated that as individuals, humans are prone to biased decision making (Kahneman, 2011) or ‘heuristic traps’ leading to accidents (McCammon, 2004), the complexity is further increased, and not well understood in research, when decision making emerges in group setting (Zweifel and Haegeli, 2014) in complex natural environments. Albeit the active phase of performance (Brymer and Schweitzer, 2017) is often executed alone, participants interact as parts of communities or groups during practice, preparation, planning or in terms of back-up safety. This addresses the need to better understand the metacognitive capacities, social systems and decision making in extreme sports in order to develop effective practical implications such as affective learning designs (e.g., Headrick et al., 2015).

To avoid biases, such as fundamental attribution error (Ross, 1977), or falling into a trap of creating superficial observational lenses and definitions on extreme sports through reductionist paradigm or inadequate theoretical approaches, it is evident and crucial to form a descriptive consensus on defining characteristics of extreme sports. Ideally, a definition should be free of negative connotations or pre-assumptions and approved by researchers and practitioners. The definition proposed in this paper builds upon a conception of ‘action and adventure sports’ (Immonen et al., 2017) and defining characteristics of the extreme sport experience as indicated by the phenomenological research. Here, we argue, that what makes the difference between extreme and non-extreme sport, is the particular type of emerging experience and the ensuing changes in ways of being-in-the-world (Heidegger, 1996). This occurs when specific performances, often by facing danger or potential death, make deep existential structures visible and available to be experienced, and which is unavailable to be reached within traditional sports or non-extreme participation styles within action and adventure sports. When adopting the definition, it is worth emphasizing that researchers need to fully understand the interacting constraints of the sport in question, and carefully consider how study designs, research questions, experimental tasks or interventions maintain the coupled dynamical processes of perception and action against the situational and contextual horizon (Brunswik, 1956; Pinder et al., 2011).

## Concluding Remarks

We have argued that an ecological dynamics framework is well-suited for the study of extreme sports due to the multi-dimensional scale of analysis combined with the encompassing and descriptive definition of extreme sport as guided by recent phenomenological research. This perspective provides a holistic lens to scrutinize extreme sports as *forms of life* specific to humans, which are clearly distinguished from traditional sports or non-extreme participation styles of action and adventure sports. In addition, the notion of form of life and different levels of analysis allow us to explore extreme sports through specific features across niches or disciplines as sociocultural frames of reference (e.g., freediving as contrasted to free solo climbing) and at a more detailed individual level (i.e., experiences or learning). Thus, for a nuanced and comprehensive understanding of extreme sports, we propose that experiences and performance outcomes of individuals should be considered as embodiments of complex and dynamic, interacting individual-environment systems emerging through the composition of situational and contextual constraints.

We have emphasized that the creative exploratory behaviors of participants form a crucial and defining characteristic. This continuous ‘search’ for the meta-stable regions of the system, where ineffable experiences and creative performance outcomes emerge, is an essential merit for an activity which, in our view, can be called ‘extreme sport’. So far, it has been the phenomenological exploration, which has offered the most valuable insights on individuals’ experiences. Therefore, by understanding extreme sports as distinguished, emerging and constantly evolving niches and adopting the definition based on previous phenomenological research findings and theoretical principles of ecological dynamics, we propose extreme sports to be defined as: *emergent forms of action and adventure sports, consisting of an inimitable person-environment relationship with exquisite affordances for ultimate perception and movement experiences, leading to existential reflection and self-actualization as framed by the human form of life.*

## Author Contributions

TI, EB, KD, JL, and TJ contributed to the conceptualization, drafting the work, and background research.

## Conflict of Interest Statement

The authors declare that the research was conducted in the absence of any commercial or financial relationships that could be construed as a potential conflict of interest.
